# Neuroendocrine carcinoma in the extrahepatic biliary tract: A case report and literature review

**DOI:** 10.1097/MD.0000000000011487

**Published:** 2018-07-20

**Authors:** Liang Zhang, DaLong Wan, Li Bao, Qing Chen, HaiYang Xie, ShiGuo Xu, ShengZhang Lin

**Affiliations:** a Division of Hepatobiliary and Pancreatic Surgery, Department of Surgery, First Affiliated Hospital, School of Medicine, Zhejiang University; b NHFPC Key Laboratory of Combined Multi-Organ Transplantation; c Key Laboratory of the Diagnosis and Treatment of Organ Transplantation, CAMS; d Department of Pathology, the First Affiliated Hospital, Zhejiang University School of Medicine, Hangzhou, China.

**Keywords:** extrahepatic bile tract, management, metastasis, neuroendocrine carcinoma

## Abstract

**Rationale:**

Neuroendocrine carcinoma (NEC) arising from the extrahepatic biliary tracts (EHBTs) is rare, and thus its management and prognosis remain poorly clarified. We herein describe a case of NEC in the perihilar EHBTs, and review the literature, together with a comparison between NECs in the perihilar and distal EHBTs, to elucidate the management strategy and oncological outcome of this rare entity.

**Patient concerns:**

A 62-year-old Chinese male was admitted with complaints of painless jaundice. Imaging studies revealed a 2-cm mass in the hepatic hilum, regional lymph node involvement, and severe stenosis at the junction of the common hepatic ducts.

**Diagnoses:**

The histopathological examination of the resected specimen demonstrated small tumor cells with round hperchromatic nuclei and scant cytoplasm. A detailed immunohistochemical analysis showed that the tumor was strongly positive for synaptophysin, CD56 and chromogranin A, with a Ki-67 labeling index greater than 80%. These results led to a diagnosis of NEC in the perihilar bile duct.

**Interventions:**

The patient underwent surgical resection including a left hemihepatectomy, cholecystectomy, lymphadenectomy and Roux-en-Y hepaticojejunostomy.

**Outcomes:**

During the two months of follow-up, repeated imaging studies indicated tumor recurrence in the liver. The patient died 6 months after surgery.

**Lessons:**

NEC in the EHBTs is extremely challenging to diagnose preoperatively because of mimicking other bile duct cancers. The prognosis of this disease entity is dismal, and most patients die within 2 years after diagnosis. Subtyping of NECs into perihilar NECs and distal NECs is beneficial for clinical applications, including guiding therapy selection and predicting survival.

## 1 Introduction

Derived from neuroendocrine cells, neuroendocrine neoplasms (NENs) are a group of heterogeneous tumors with variable clinical and pathologic features.^[1]^ Some are indolent, while others show a great tendency to spread, depending upon the histologic differentiation and sites of origin. Given this context, NENs are currently classified as neuroendocrine tumors (NETs) and neuroendocrine carcinomas (NECs).^[2]^ NECs are defined as malignant NENs with poor differentiation and high proliferation rates (Ki-67 > 20% and/or mitotic count >20 per 10 high-power fields). Accordingly, NECs show an increased tendency for distant metastasis, leading to a poor prognosis.^[3]^

The NECs are predominantly located in the lung and gastrointestinal tract.^[1,4]^ Occasionally, NECs occur in the biliary system, with the gallbladder representing the most common site.^[5]^ NECs in the extrahepatic biliary tract (EHBT) are extremely rare, with a total of 21 cases reported in the English literature, and thus its management strategy and oncologic outcome remain poorly clarified.

The EHBT refers the bile tract outside the liver and extending to the level of the ampulla. Separated by the cystic duct, EHBTs are classified into perihilar and distal EHBT.^[6]^ The most common malignancy of the EHBTs is cholangiocarcinoma (CCA), which accounts for 90% of cancers in this location.^[7]^ It is well-established that perihilar CCA (pCCA) and distal CCA (dCCA) have distinct biologic and epidemiologic characteristics, diagnostic and therapeutic approaches, and even prognoses.^[8]^ Thus, it is rational to hypothesize that certain distinctions also exist between NECs in the perihilar and distal EHBT.

For a better established recognition of this rare entity, we herein present a case of an NEC of the hilar bile duct in a 62-year-old Chinese male and a literature review of previously reported cases. Furthermore, despite the paucity of cases, we sought to compare the clinicopathologic characteristics of perihilar NEC (pNEC) and distal NEC (dNEC).

## 2 Case report

**Figure 1:**
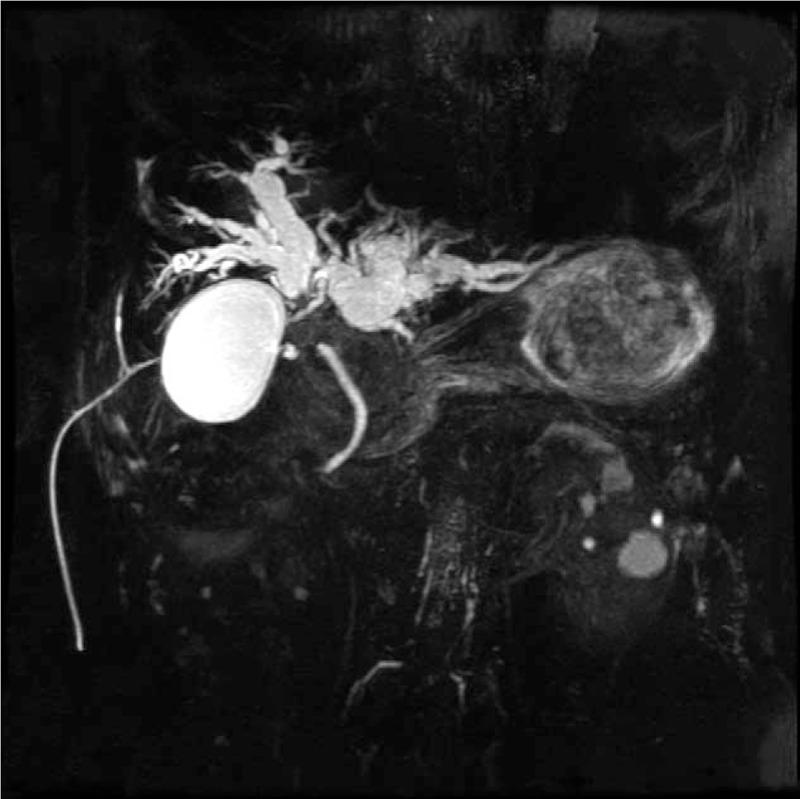
Magnetic resonance cholangiopancreatography showed severe stenosis at the junction of the left and right common hepatic ducts and marked dilation of the upstream bile duct.

**Figure 2:**
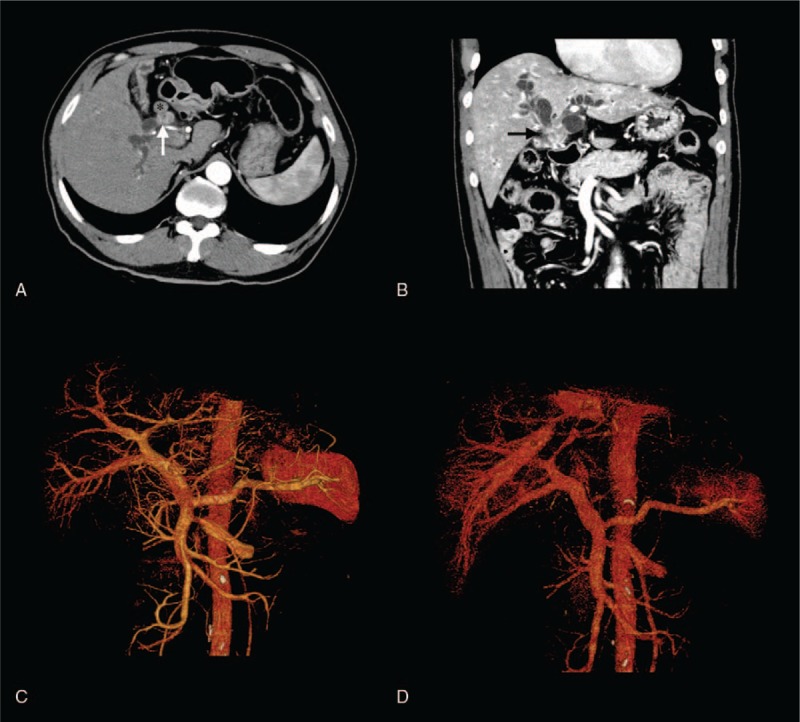
Abdominal enhanced computed tomography (CT) images. (A, B) An enhanced CT scan showed a moderately enhanced mass located in the hilar bile duct (white and black arrows) with regional lymph node involvement (asterisk). (C, D) CT angiography and 3-dimensional reconstruction revealed the absence of vascular invasion.

A 62-year-old Chinese male was referred to our center with complaints of painless jaundice. He had no family history of cancer. His medical history was significant for pulmonary tuberculosis, which had been treated with medications. On admission, a physical examination showed moderately icteric sclera and jaundice. The circulatory, respiratory, and abdominal examinations were unremarkable. The initial laboratory tests revealed the following: elevated total bilirubin, 403 μmol/L (normal, 0–21 μmol/L); aspartate aminotransaminase, 153 U/L (normal, 5–40 U/L); alanine aminophosphatase, 93 U/L (normal, 8–40 U/L); carcinoembryonic antigen (CEA), 10.2 ng/mL (normal, 0–5 ng/mL), and carbohydrate antigen 19-9 (CA19-9), 1073.6 U/mL (normal 0–35 U/mL). A pulmonary computed tomography (CT) scan was negative except for a few areas of fibrosis resulting from the previous tuberculosis infection. Magnetic resonance cholangiopancreatography showed severe stenosis at the junction of the left and right common hepatic ducts and marked dilation of the intrahepatic bile ducts (Fig. 1). An abdominal enhanced CT scan revealed a 2-cm, moderately enhanced mass in the hepatic hilum and regional lymph node enlargement (Fig. 2). Percutaneous transhepatic biliary drainage was performed to relieve cholestasis and improve liver function. With a tentative diagnosis of pCCA (Bismuth IV type), the patient underwent surgical resection including a left hemihepatectomy, cholecystectomy, and lymphadenectomy. The reconstruction was achieved by Roux-en-Y hepaticojejunostomy. This procedure was considered curative since intraoperative frozen examination showed that the resection margin was free of atypical cells.

**Figure 3:**
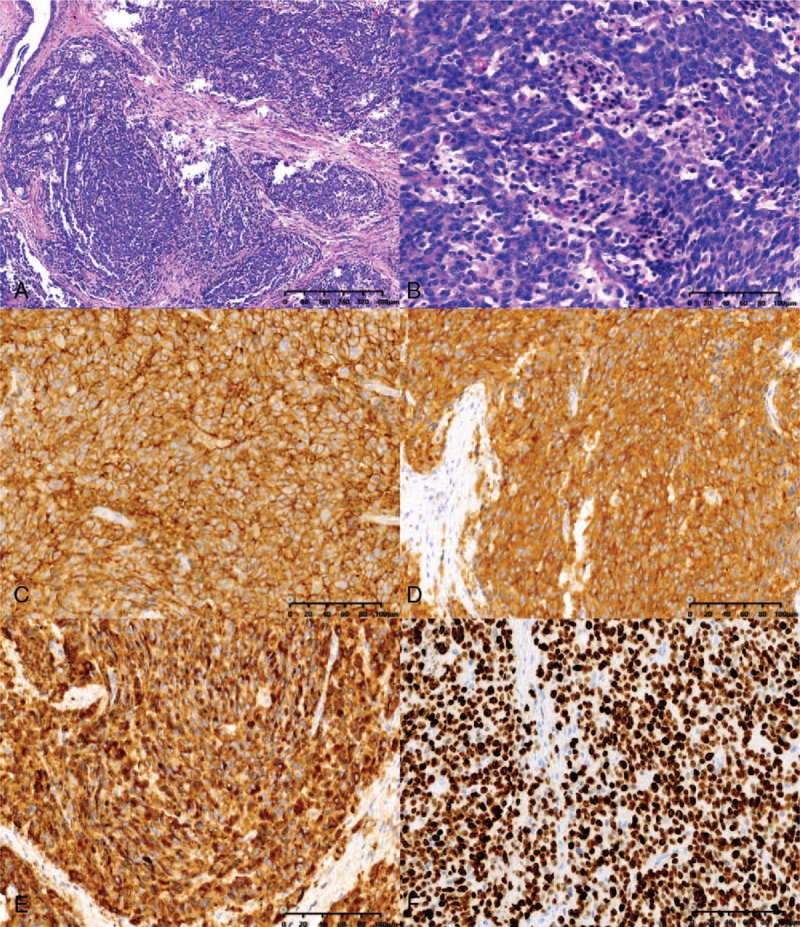
Microscopy images of the surgical specimen. (A) The tumor showed a nested organoid growth pattern (hematoxylin and eosin [HE], ×100). (B) The tumor cells were small in size and had round, hyperchromatic nuclei and scant cytoplasm (HE, ×400); immunohistochemical examinations revealed that the tumor cells were positive for CD56 (C, ×400), synaptophysin (D, ×400), and chromogranin (E, ×400); more than 80% of the tumor cells were positive for Ki-67 (F, ×400).

Within the resected specimen, a yellowish tumor measuring 2 cm × 0.5 cm × 1 cm was found in the hilar bile duct. Microscopically, the tumor showed a nested organoid growth pattern. The tumor cells were small in size and had round hyperchromatic nuclei and scant cytoplasm (Fig. 3A, B). Metastasis was detected in 1 of 3 resected hepatoduodenal ligament lymph nodes and 2 of 2 cystic lymph nodes. A detailed immunohistochemical (IHC) analysis confirmed a highly proliferative NEC that was chromogranin A (+), CD56 (+), synaptophysin (+), and Ki-67 (+, >80%) (Fig. 3C–F).

The postoperative course was complicated. The patient developed prolonged bile leakage, and adjuvant chemotherapy was postponed. Repeat abdominal imaging 2 months after the initial diagnosis showed tumor recurrence in the right liver lobe, and the patient died 6 months after the operation.

## 3 Discussion

The NENs are epithelial neoplasms that demonstrate neuroendocrine phenotype, including the synaptic-like vesicles, secretory granules, and production of amine hormones.^[9]^ Only 0.32% of NENs occur in the EHBT, and NETs represent the most common type of tumor.^[5,10]^ Michalopoulos et al have collected a total of 150 cases of NETs in the EHBT in the literature, and concluded that NETs in the EHBT were difficult to diagnose preoperatively while associated with favorable prognosis.^[11]^ Distinct from NETs, NECs are poorly differentiated and extremely aggressive malignancies.^[1]^ NECs in the EHBT are exceedingly rare and are not well elucidated.

We searched PubMed, Google Scholar, and Web of Science for English language reports describing NECs in the EHBT. Only 21 cases were reported after excluding NECs in the intrahepatic bile duct, gallbladder, and ampulla of Vater.^[12–31]^ We briefly summarized the major clinical and pathologic features (Table 1) and compared the features of pNEC and dNEC (Table 2).

**Table 1:**
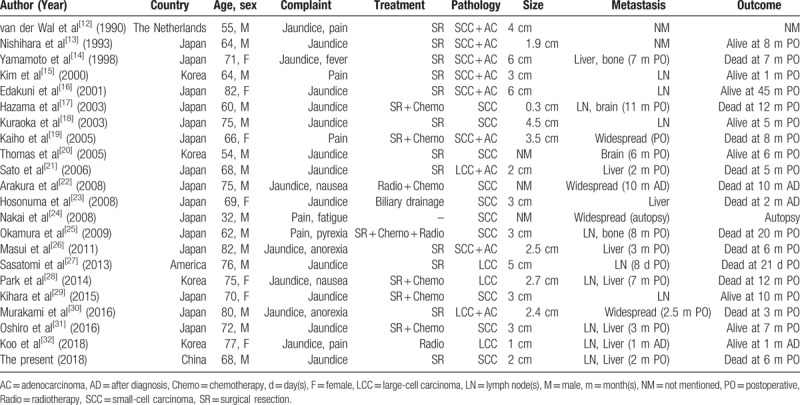
Summary of cases of neuroendocrine carcinomas in the extrahepatic biliary tract.

**Table 2:**
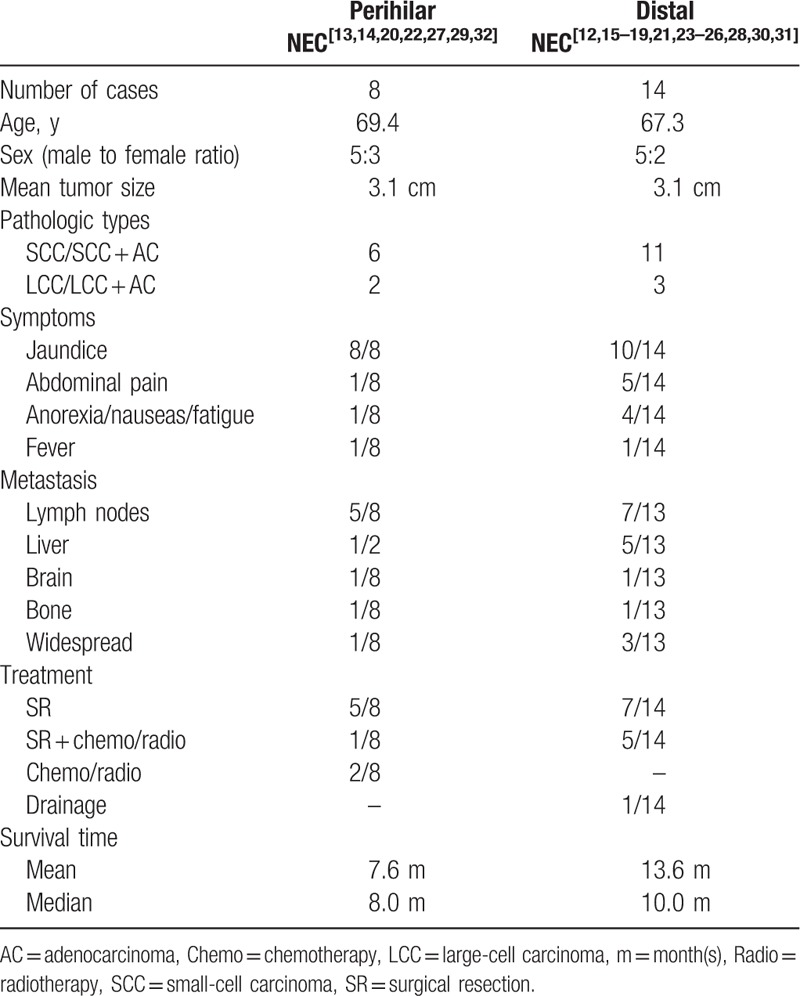
A comparison of neuroendocrine carcinomas (NECs) in the perihilar and distal extrahepatic biliary tract.

Of the 22 cases of NECs in the EHBT (including the present case), the mean age of the patients was 68.0 years (range 32–82 years). Males predominated with a male: female ratio of 15:7. Concurrent *Clonorchis sinensis* infection was observed in 2 patients from Korea.^[15,20]^ The tumor size ranged from 0.3 to 6.0 cm with a mean size of 3.1 cm. Presentation symptoms of NECs in the EHBT were mostly due to the effects of the mass and included obstructive jaundice (18/22 patients), abdominal pain (6/22 patients), anorexia/nausea/fatigue (5/22 patients), and fever (2/22 patients). Compared with dNECs, pNECs were less common, with only 8 cases reported.^[13,14,20,22,27,29,32]^ Patients with pNEC exhibited abdominal pain less frequently. Other baseline characteristics, including age, gender, and tumor size, did not significantly differ between the 2 entities.

Laboratory findings of biliary NECs usually highlight elevated serum levels of CEA and CA19-9, which are classical but nonspecific biomarkers of bile duct tumors. Serum hormone levels are mostly within normal limits, in contrast to other types of NECs, such as pancreatic or bronchial NECs, that are frequently associated with hormone production.^[33]^ The absence of elevated hormone levels and corresponding hormonal symptoms suggests that biliary NECs tend to be functionally indolent.^[34]^ However, these features may discourage clinicians from suspecting a diagnosis of NEN.

Radiologic techniques generally fail to distinguish NECs from other bile duct cancers because of their overlapped imaging appearance, which contributes to the high rate of misdiagnosis. On CT, NECs typically appear as solid, homogeneously hypo-intense, slightly, or moderately enhanced masses. Cholangiography often yields stenosis or an asymmetric filling defect in the EHBT. NECs could also exhibit an abnormal high uptake of ^18^F-fluorodeoxyglucose on positron emission tomography.^[30]^ Upstream bile duct dilations, lymph node involvement and adjacent stricture invasions are frequently observed on imaging. Similar to previous cases of NECs in the EHBT, our patient displayed obstructive jaundice, elevated serum CEA and CA19-9, and without hormone-related signs or specific imaging features.

Histologic biopsy may carry a high false negative rate. In a review of reports, brush cytology was performed in 6 cases, and an NEC was detected in only 1 case.^[20]^ Arakura et al reported a case of pNEC in which the patient received endoscopic ultrasound-guided fine-needle aspiration (EUS-FNA) for diagnosis.^[22]^ The author concluded that EUS-FNA facilitated diagnosis for this entity. However, EUS-FNA is generally not recommended for patients with perihilar bile duct cancers because of the high risk of tumor seeding (83%).^[35]^ Taken together, in most cases, a definitive diagnosis cannot be made until postoperative pathology or autopsy results are available.

Pathologic and IHC investigations are usually required for a definitive diagnosis of biliary NECs. Macroscopically, typical findings of NECs include a whitish or yellowish, solid mass, and thickened bile duct walls. Microscopically, NECs are traditionally divided into small-cell carcinoma (SCC) and large-cell carcinoma (LCC) on the basis of cytologic morphology. SCC is typically characterized by small, round, atypical cells, with hyperchromatic nuclei, and scant cytoplasm. These cytologic features are unique and similar to pulmonary SCC. Only 5 cases of LCC have been reported in the literature.^[21,27,28,30,32]^ Compared to SCC, LCC tumor cells are larger in size and have a lower nuclear to cytoplasmic ratio. IHC analysis is required to confirm histologic identification. A panel of 3 IHC staining markers, chromogranin, synaptophysin, and CD56, is used to support the diagnosis of NEC and distinguish it from adenocarcinoma.^[36]^ Uccella et al suggested a combined expression of chromogranin and synaptophysin was adequate to confirm neuroendocrine differentiation.^[9]^ Although it is not a neuroendocrine marker, Ki-67 is crucial for the proliferative activity assessment and distinction of NECs from NETs.

The NEC is typically a devastating malignancy with an increased tendency for metastasis. It is estimated that over half of patients with gastroenteropancreatic NECs exhibit distant metastasis at the initial diagnosis.^[34]^ NECs in the EHBT are relatively aggressive. In our review, regional lymph node invasion was noted in 12 patients on initial admission, and distant metastasis occurred in 16 cases. dNEC and pNEC most commonly metastasize to the liver, followed by the brain and bone. Notably, dNEC and pNEC both spread rapidly; all metastases occurred within 1 year after surgery (ranging from 8 days to 11 months postoperatively). Furthermore, multimodal treatment appeared to be ineffective in delaying metastatic events.

The experience of clinicians in the treatments of NECs in the EHBT is greatly limited because of its rarity. In these reported cases, surgery was the main treatment (performed in 18/22 patients, 82%) and was regarded as the only curative option. To obtain adequate oncologic margins, extensive radical excisions are generally required for biliary NECs, similar to CCAs or other NECs.^[37,38]^ The surgical strategy of NECs in the EHBT depends greatly on the tumor location. Resections of pNEC usually involved lobectomy with bile duct resection, lymph node dissection, and Roux-en-Y hepaticojejunostomy. Surgical resection of dNEC frequently involved pancreatoduodenectomy.

The role of adjuvant therapies in biliary NECs remains largely undetermined. Due to the lack of standard indications, chemotherapy was employed in 7 cases, with the intent to help improve resectability or control tumor progression.^[25,31]^ Extrapolating from pulmonary NECs, chemotherapy regimens for biliary NECs commonly consist of cisplatin and etoposide.^[37]^ However, the effectiveness does not appear to be promising; evolving data show that the response rates are much lower in patients with extrapulmonary NECs than in those with pulmonary NECs, and NECs in the hepatic-biliary-pancreatic systems have the worst response rates, as well as relatively severe toxicity.^[37]^ In cases of tumor recurrence or metastasis, radiotherapy should be considered.^[32,34]^ Liver transplantation, a potentially curative option for a subset of patients with localized pCCA, may also be an appealing option in select cases of pNEC.^[39,40]^

A worse survival outcome is observed when NECs are primarily located in the lung, gastrointestinal tracts, and hepatic-biliary-pancreatic systems, in decreasing order, despite sharing similar histology.^[3,41]^ For NEC in the EHBT, the prognosis is extremely dismal. In the 17 reported cases in which surgeries were performed, the survival duration ranged from 21 days to 45 months. In the patient who had the longest survival, the Ki-67 labeling index was <10%, which indicates that, according to the current World Health Organization (WHO) classification system, the appropriate diagnosis was NET rather than NEC.^[16]^ This correct distinction makes the longest survival time 23 months rather than 45 months, which is particularly disappointing.^[25]^ Regarding the remaining 4 patients treated without surgical intervention, none survived more than one year. A Kaplan–Meier analysis of 19 patients with survival data was performed, which showed that survival was worse in patients with pNEC than in those with dNEC; the mean survival times of pNEC and dNEC were 7.6 months and 13.6 months, respectively, although this difference was not statistically significant. The postoperative survival appeared slightly worse in our case (6 months) when compared with the previous cases, presumably due to the combination of the highly invasive tumor biology, as measured by a high Ki-67 index (>80%), and the unfavorable tumor location (pNEC) in our patient.

One of the major limitations of the present study is the small number of cases available for analysis. However, this is inevitable due to the rarity of the disease entity. Additional reported cases are needed to establish a clearer consensus on the management and prognosis of NECs in the EHBT.

## 4 Conclusion

Establishment of a preoperative diagnosis of NEC in the EHBT is extremely challenging due to the tendency of NECs to mimic other bile duct cancers, in addition to the rarity of this type of cancer. Patients with this entity have extremely dismal survival times due to its aggressive course, and most die within 2 years after diagnosis. Analogous to CCA, we suggest that subtyping of NECs into pNEC and dNEC is beneficial for clinical applications, including guiding therapy selection and survival prediction.

## Acknowledgments

The authors acknowledge the support of the Department of Radiology, the First Affiliated Hospital, Zhejiang University School of Medicine.

## Author contributions

**Conceptualization:** Liang Zhang, ShengZhang Lin.

**Data curation:** Qing Chen.

**Investigation:** Qing Chen.

**Methodology:** DaLong Wan.

**Supervision:** ShengZhang Lin.

**Validation:** ShengZhang Lin.

**Visualization:** HaiYang Xie, ShiGuo Xu.

**Writing – original draft:** Liang Zhang, DaLong Wan, Li Bao.

**Writing – review & editing:** HaiYang Xie, ShiGuo Xu, ShengZhang Lin.
